# A Study on Sensitivity Improvement of the Fiber Optic Acoustic Sensing System Based on Sagnac Interference

**DOI:** 10.3390/s24196188

**Published:** 2024-09-24

**Authors:** Ruixi Tang, Hongcheng Zhao, Juqin Feng, Jiang Wang, Ning Wang, Jun Ruan, Jianjun Chen

**Affiliations:** 1 The Key Laboratory of Electromagnetic Technology and Engineering, Nanchong of Sichuan, China West Normal University, Nanchong 637000, China; tangruixi@stu.cwnu.edu.cn (R.T.); 834806354@stu.cwnu.edu.cn (H.Z.); windcola@stu.cwnu.edu.cn (J.F.); ruanj2005@163.com (J.R.); 2 The Institute of Xi’an Aerospace Solid Propulsion Technology, Xi’an 710025, China; cquwj313@163.com; 3 The Key Laboratory of Optoelectronic Technology & System, Education Ministry of China, Chongqing University, Chongqing 400044, China; ningw@cqu.edu.cn

**Keywords:** acoustic sensing system, Sagnac, pickup optimization, grooves

## Abstract

A new pickup structure was introduced and modified to improve the resolution of the linear Sagnac optical fiber acoustic sensing system. The maximum strains corresponding to the material, diameter, wall thickness, and height of the pickups were analyzed by simulation. An aluminum cylinder with a diameter of 110 mm, a wall thickness of 3 mm, and a height of 120 mm was chosen as the basic pickup. A four-groove pickup with a vertical width of 80 mm and a horizontal width of 20 mm was introduced to improve the sensitivity of the system. The experiments showed that the average peak-to-peak sensitivity of the four-groove pickup increased by 215.54% to 106.806 mV/Pa. The improved pickup can be applied in areas to monitor the situation of invasion of the Sagnac optical fiber acoustic sensing system.

## 1. Introduction

The fiber optic acoustic sensing system is widely used in perimeter security, infrastructure inspection, and healthcare due to a small size, light weight, high sensitivity, and good environmental tolerance [1,2,3,4,5,6]. According to principles and signal detection methods, distributed fiber optic sensors are classified into sensors based on light backward scattering and sensors based on a fiber optic interference structure. Sensors based on light backscattering are classified into optical time domain reflectance (OTDR) sensors, optical frequency domain reflectance (OFDR) sensors, etc. Sensors based on a fiber optic interference structure can be classified as Mach–Zehnder, Michelson, Fabry–Perot, and Sagnac sensors [7,8,9,10,11,12]. The Sagnac fiber optic acoustic sensing system has the advantages of optical reciprocity, high sensitivity, and strong adaptability to the environment, which is widely applied in the harsh environment in the field [13,14,15].

Shao et al. enhanced the sensitivity by splicing a section of bias-preserving fiber between two standard single-mode fibers and bending the structure into a circle. Wang et al. proposed a linear Sagnac fiber optic acoustic sensing system. The influence of the delay fiber and sensing fiber on the system was studied by theoretical calculation and simulation analysis, and the optimal length of the delay fiber and sensing fiber was determined, with a sensitivity of 30.67 mV/Pa. Chen et al. proposed an improved pickup structure to enhance the sound recognition sensitivity of a linear Sagnac fiber optic acoustic sensing system by phase change, which increased the sensitivity by 71.2% between 100 and 1500 Hz [16,17,18]. In summary, most of the studies have focused on improving the sensitivity of fiber optic acoustic sensing systems in different ways. In this paper, a new pickup was proposed to improve sensitivity, and comparative experiments were carried out.

## 2. Structural Analysis

### 2.1. Linear Sagnac Fiber Optic Acoustic Sensing System

**Figure 1:**
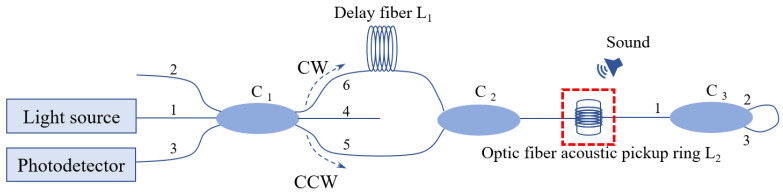
Linear Sagnac fiber optic acoustic sensing system.

As shown in Figure 1, light enters the optical path from port 1 of the 3 × 3 coupler C_1_ and is divided into two beams, which are output from ports 5 and 6, respectively. The light of the clockwise output from port 6 passes through the delay fiber L_1_ to the 2 × 1 coupler C_2_. The light of the counterclockwise output from port 5 passes directly into C_2_, which combines the two beams into one. The beam passes through the pickup fiber ring L_2_ and to port 1 of the 1 × 2 coupler C_3_. Next, the beam output from port 2 or 3 of C_3_ re-enters C_3_ from the other port and returns along the optical path from port 1 of C_3_. Interference occurs at C_1_, and the interference light is detected by a photodetector.

### 2.2. Pickup Structure Analysis

**Figure 2:**
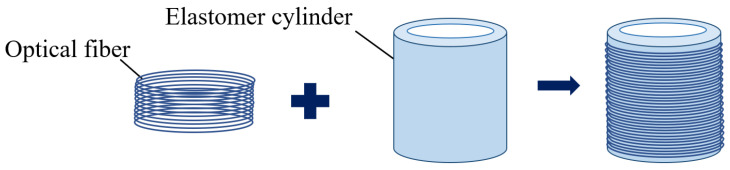
The structure of the pickup.

It is difficult to realize high-resolution sound detection with optical fiber as the sensitive element of the sensing system. As shown in Figure 2, the structure of the fiber wound to the outer wall of the elastomer cylinder was proposed. The sensitivity of the system was improved by “amplifying” the effect of sound, which increased the phase change of light waves in the optical fiber.

**Figure 3:**
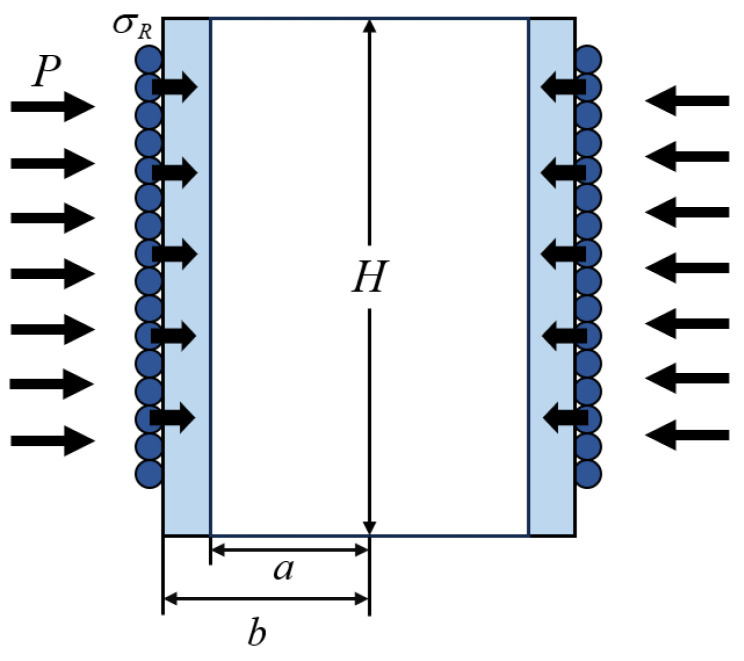
The schematic diagram of the boundary conditions of the pickup.

As shown in Figure 3, the inner and outer walls of the pickup were affected by the acoustic pressure, *P*. The outer wall of the pickup was tightly wound with optical fiber, and the reaction force was $\sigma_{R}$.

The boundary conditions can be expressed as

(1) $$\left\{\begin{array}{l} \sigma_{r|r=a}=-P\\ \sigma_{r|r=b}=-(\sigma_{R}+P)\\ \sigma_{z}=\sigma_{\theta }=0 \end{array}\right.$$

where *a* is the inner diameter and *b* is the outer diameter. Δr is the radial displacement of the pickup. It can be expressed as [19]

(2) $$\begin{array}{cc} \Delta r & =u_{r=b}\\ & =C_{1}b+\frac{C_{2}}{b}\\ & =-\frac{(1+\mu )(1-2\mu )(\mu -1)b^{2}}{(2\mu^{2}+\mu -1)(b^{2}-a^{2})E}(\sigma_{R}+\frac{b^{2}+a^{2}}{b^{2}}P)\\ & -\frac{(1+\mu )a^{2}b}{(b^{2}-a^{2})E}\left[\sigma_{R}-\frac{\left(b^{2}-a^{2})(b^{2}-1\right)}{b^{2}}P\right] \end{array}$$

where μ is the Poisson’s ratio of the fiber and *E* is the modulus of elasticity of the fiber.

Let

(3) $$\left\{\begin{array}{l} O_{1}=(2\mu^{2}+\mu -1)(1+\mu )\\ O_{2}=(1+\mu )(1-2\mu )(\mu -1)\\ O_{3}=2\mu^{2}+\mu -1\\ N=\frac{L}{2\pi b}\\ H=Nd_{f}=\frac{Ld_{f}}{2\pi b} \end{array}\right.$$

(4) $$\left\{\begin{array}{l} C_{1}=-\frac{(1-\mu )(1-2\mu )(\mu -1)b^{2}}{(2\mu^{2}+\mu -1)(b^{2}-a^{2})E}(\sigma_{R}+\frac{b^{2}+a^{2}}{b^{2}}P)\\ C_{2}=-\frac{(1+\mu )a^{2}}{(b^{2}-a^{2})E}\left[\sigma_{R}-\frac{\left(b^{2}-a^{2})(b^{2}-1\right)}{b^{2}}P\right]\\ C_{3}=-\frac{2\mu (1-\mu )(1-2\mu )b^{2}}{(2\mu^{2}+\mu -1)(b^{2}-a^{2})E}(\sigma_{R}+\frac{b^{2}+a^{2}}{b^{2}}P) \end{array}\right.$$

Let $k_{f}=E_{f}S_{f}$, where $E_{f}$ is the elastic modulus and $S_{f}$ is the cross-sectional area of the fiber. *N* is the number of coils of fiber wrapped around the pickup. *H* is the effective height of the wound optical fiber.

Let $m_{1}=-\frac{\left[O_{1}(b^{2}-a^{2})(1-b^{2})a^{2}b^{2}+O_{2}(a^{2}+b^{2})\right]d_{f}}{EO_{3}(b^{2}-a^{2})b^{3}d_{f}+\left(O_{1}a^{2}b^{2}+O_{2}\right)k_{f}}$. When the optical fiber is placed in the sound field, the length is affected by the sound pressure, which causes a phase change. It can be formulated as [17]

(5) $$\Delta \varphi_{s}=\beta \Delta L_{s}=\beta a2\pi \Delta r=\frac{\beta Lam_{1}P}{b}$$

where *a* is an empirical value, taken to be 0.4.

(6) $$\begin{array}{cc} \Delta \varphi & =\Delta \varphi_{L}+\Delta \varphi_{n}+\Delta \varphi_{s}\\ & =\beta Lk_{1}P+\frac{\beta Lm_{1}aP}{b}\\ & =\beta L\left(k_{1}+\frac{m_{1}a}{b}\right)P \end{array}$$

Equation (6) shows that the phase change of the optical signal increases with the introduction of the elastomer cylinder, which improves the detection sensitivity of the system.

**Figure 4:**
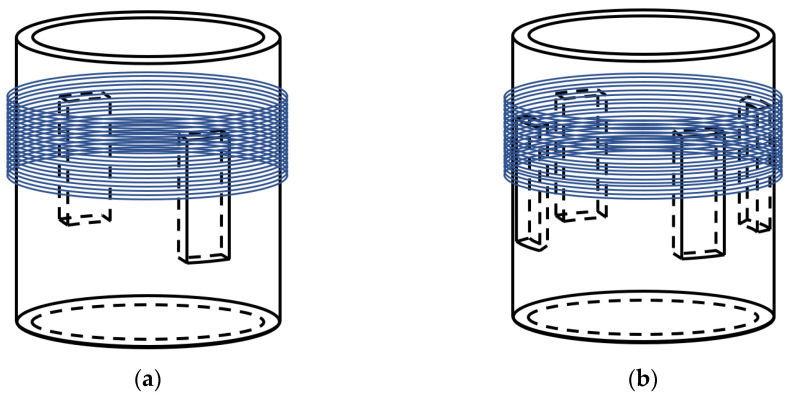
The structure of the improved pickups: (**a**) the pickup with two grooves and (**b**) the pickup with four grooves.

Slotting in elastomers is a potential method to improve the response of the pickup to the sound signal [20], and grooved pickups were proposed to improve the sound response sensitivity in this paper. The structure diagram of the slotted pickups is shown in Figure 4. Rectangular grooves were evenly distributed in the elastomer cylinder. The optical fiber was evenly wound in the slot of the elastomer cylinder to form the improved pickup. A finite element model of the improve pickup was developed, and the simulation was finished based on Solidworks.

## 3. Simulations

### 3.1. The Basic Elastomer Cylinder

**Figure 5:**
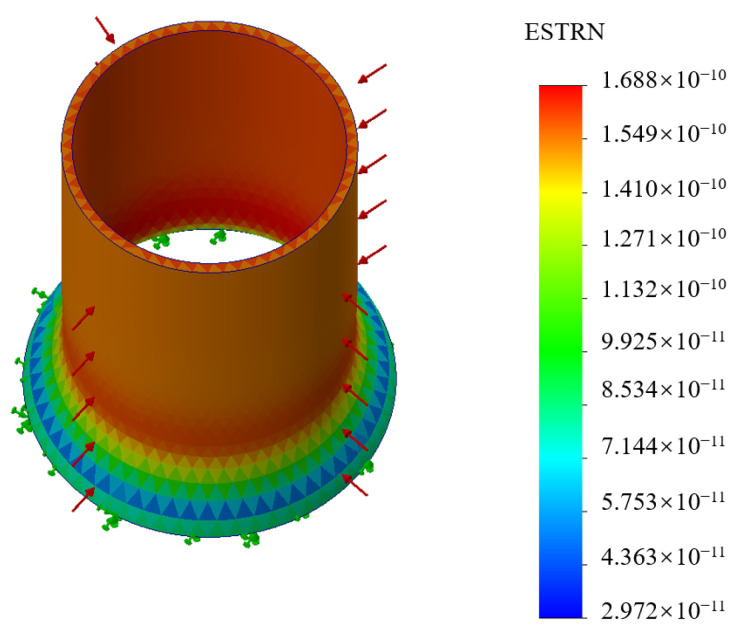
The analysis of the maximum strain.

As shown in Figure 5, the model of the elastomer cylinder was constructed by Solidworks. With an external force of 1 Pa, the bottom of the elastomer cylinder was fixed, and the maximum strain was analyzed with different materials, diameters, wall thicknesses, and heights.

Table 1 shows the simulation results of different materials. The maximum strain of aluminum was the largest among these five materials, which was 1.706 × 10^−10^. The aluminum elastomer cylinder was more conducive to improve the system resolution.

**Figure 6:**
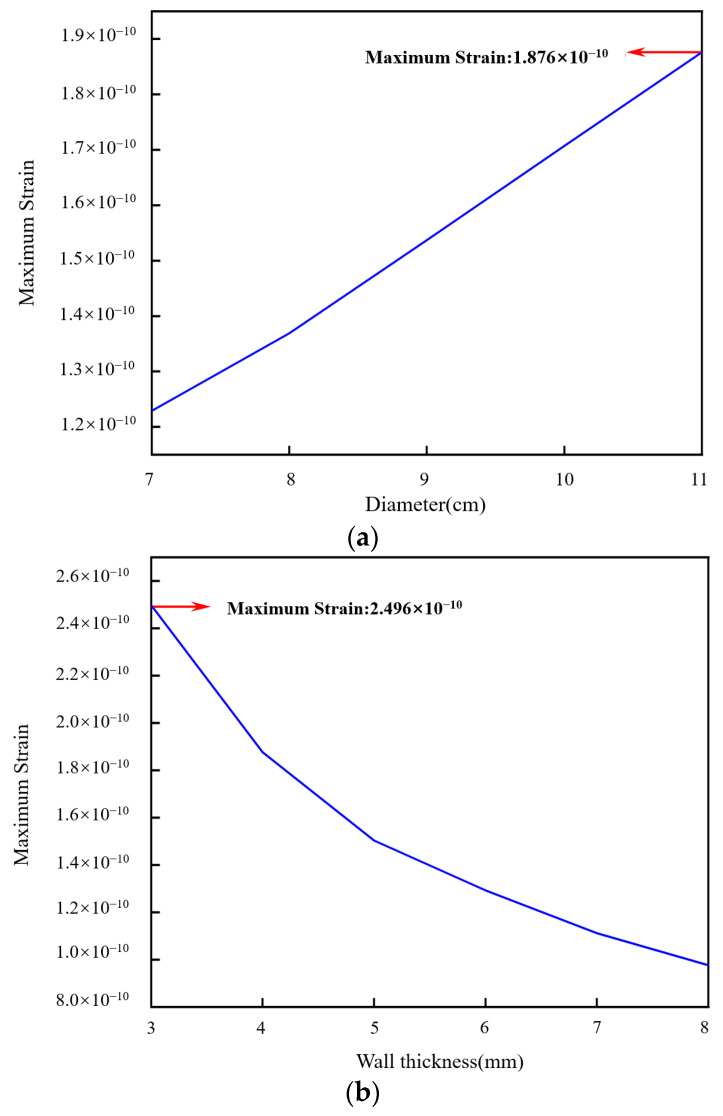

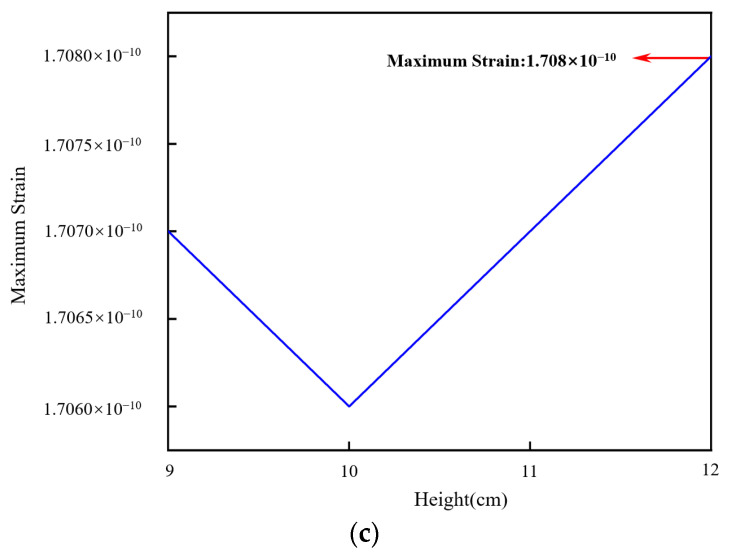
The maximum strain of the aluminum cylinder: (**a**) the maximum strain with the diameter, (**b**) the maximum strain with the wall thickness, and (**c**) the maximum strain with the height.

Figure 6a–c shows the maximum strain corresponding to the aluminum cylinder with a diameter of 7–11 cm, a wall thickness of 3–8 mm, and a height of 9–12 cm, respectively. The simulation interval of the diameter was 1 cm, the wall thickness was 1 mm, and the height was 1 cm. The range of diameters, wall thicknesses, and heights were selected based on the installation dimensions in the actual application. As shown in Figure 6a, the maximum strain of the pickup increased with the diameter at the same sound pressure. The maximum strain reached 1.876 × 10^−10^ when the diameter was 11 cm. As shown in Figure 6b, the maximum strain of the pickup decreased with the wall thickness at the same sound pressure. The maximum strain was 2.496 × 10^−10^ with a wall thickness of 3 mm. As shown in Figure 6c, the maximum strain varied slightly with the height at the same sound pressure. The maximum strain was 1.708 × 10^−10^ when the height was 12 cm. The aluminum cylinder with a diameter of 11 cm, a wall thickness of 3 mm, and a height of 12 cm was chosen as the basic cylinder.

**Figure 7:**
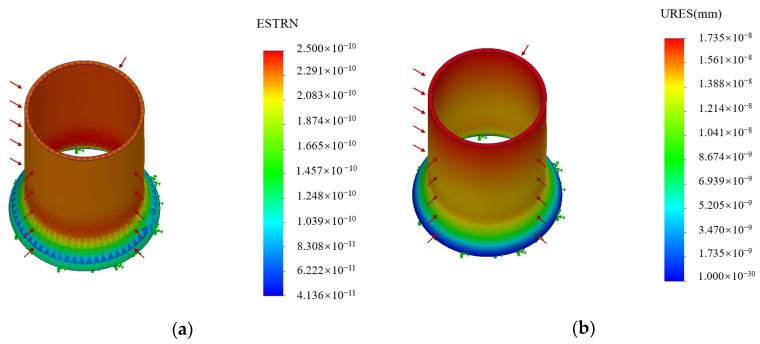
The static stress analysis of the aluminum cylinder: (**a**) the maximum strain of the aluminum cylinder and (**b**) the maximum displacement of the aluminum cylinder.

Figure 7 shows the maximum strain and maximum displacement of the aluminum cylinder. The maximum strain was 2.500 × 10^−10^, and the maximum displacement was 1.735 × 10^−8^ mm. The results showed that the closer the optical fiber to the top of the elastomer cylinder, the greater the strain and displacement. The optical fiber was wound close to the upper part of the elastomer cylinder in subsequent experiments.

### 3.2. Improved Elastomer Cylinder

**Figure 8:**
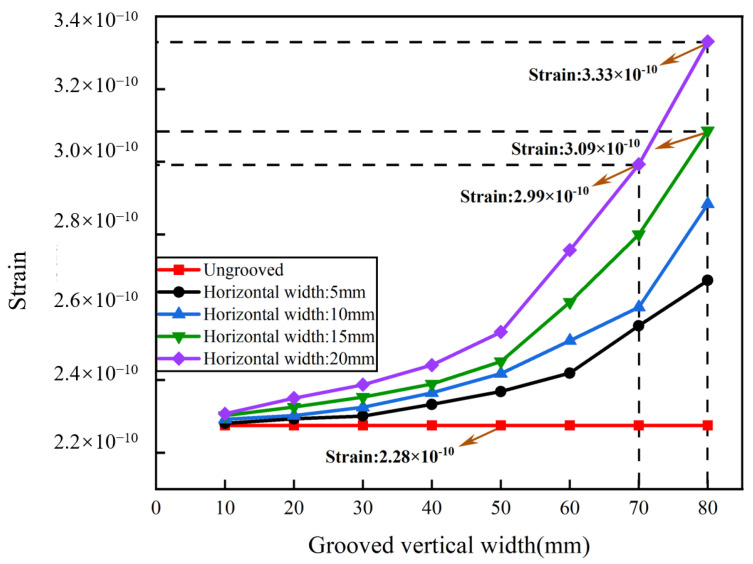
Strain analysis with two grooves.

**Figure 9:**
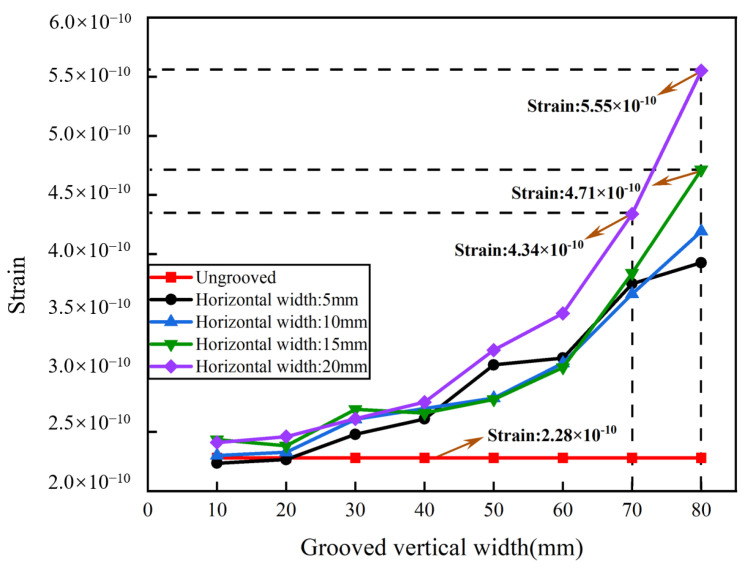
Strain analysis with four grooves.

**Figure 10:**
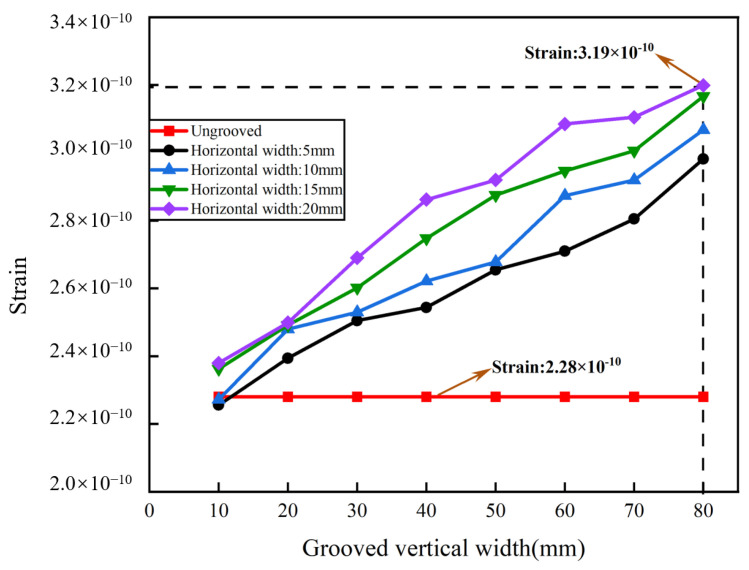
Strain analysis with six grooves.

The basic aluminum cylinder was optimized by grooving to improve the sensitivity of the sensing system. Two-groove, four-groove, and six-groove pickup models were constructed. The bottom of the pickup was fixed in the experiment, and the central position of the outer wall of the cylinder was selected for grooving. As the optical fiber was wrapped around the outer wall of the cylinders, the size of the grooves was not oversized. The groove size of the cylinder was set to be 10–80 mm in vertical width and 5–20 mm in horizontal width. The interval of the vertical width was 10 mm, and the interval of the horizontal width was 5 mm. With the same groove dimensions of 80 mm vertical width and 20 mm horizontal width, the maximum strain of the two grooves reached 3.33 × 10^−10^, the maximum strain of the six grooves reached 3.19 × 10^−10^, and the maximum strain of the four grooves reached 5.55 × 10^−10^. Figure 8 shows that the strain of the two-groove pickup increased with the vertical width and the horizontal width. The strain reached 3.33 × 10^−10^ when the vertical width was 80 mm and the horizontal was 20 mm, which was improved by 46.05% compared to the ungrooved pickup. As shown in Figure 9, the strain of the four-groove pickup increased with the vertical width and the horizontal width. The strain was 4.34 × 10^−10^ when the vertical width was 70 mm and the horizontal was 20 mm, which was improved by 90.35% compared to the ungrooved pickup. The strain was 4.71 × 10^−10^ when the vertical width was 80 mm and the horizontal was 15 mm, which was improved by 106.58% compared to the ungrooved pickup. The strain was 5.55 × 10^−10^ when the vertical width was 80 mm and the horizontal was 20 mm, which was significantly improved by 143.42% compared to the ungrooved pickup. Figure 10 shows that the strain of the six-groove pickup increased with the vertical width and the horizontal width. The strain reached 3.19 × 10^−10^ when the vertical width was 80 mm and the horizontal was 20 mm, which was improved by 39.91% compared to the ungrooved pickup. The strain of the enhancement of the six-groove pickup deteriorated. The maximum strain of the pickup increased from the two-groove to the four-groove pickup and decreased from the four-groove to the six-groove pickup. The pickups with six grooves were not selected for further study in this paper. The pickups with a strain of 3.33 × 10^−10^, 4.34 × 10^−10^, 4.71 × 10^−10^, and 5.55 × 10^−10^ were selected for processing.

## 4. Experiments

**Figure 11:**
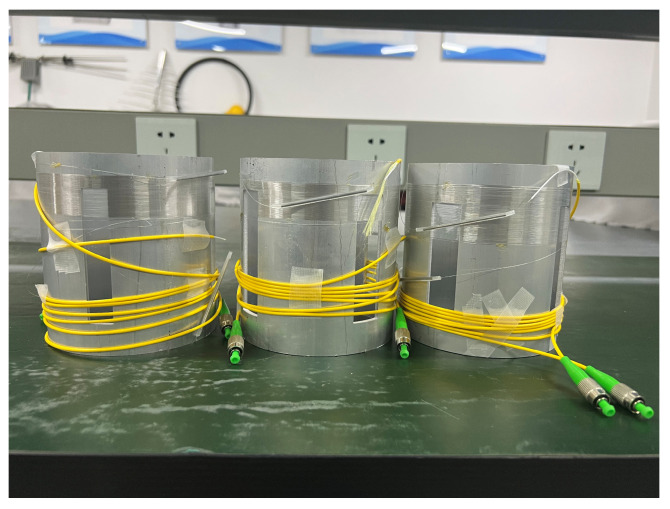
A photo of the partial pickup.

**Figure 12:**
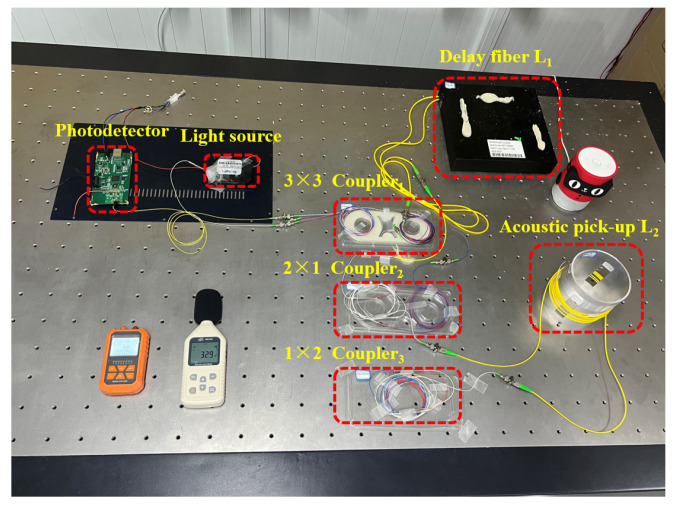
A photo of the sensing system.

The improved pickups are shown as Figure 11, and the sensing system based on an improved pickup is shown as Figure 12. The system used the SLD light source GR1346Q-A and the InGaAs detector GT322D, which were developed by the 44th Research Institute of the China Electronics Technology Group Corporation (CETC). The G652D delay fiber and single-mode optical fiber were used in the system, which are produced by Changfei Company, Shanghai, China. The length of the delay fiber is 2KM, and the shell size is 20 cm × 20 cm × 4 cm. The length of the optical fiber wrapped around the outer wall of the cylinder is 20 m. The optical fiber is uniformly and densely wound within 10–16 mm of the upper and 70–84 mm of the bottom of the elastomer cylinder. The decibel meter ensures that the sound pressure received by the pickup is constant.

**Figure 13:**
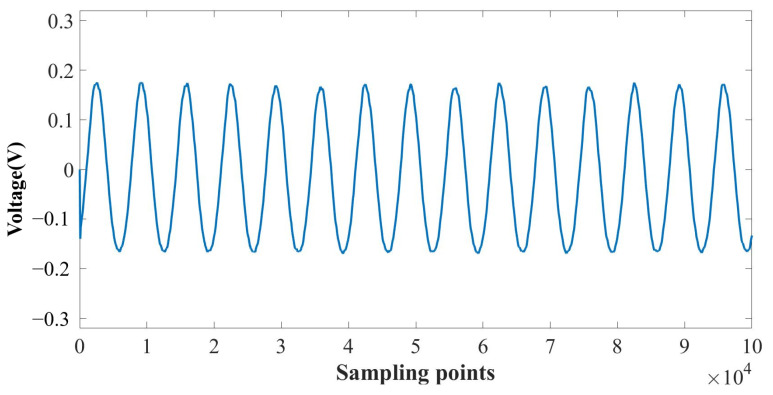
Output voltage at 3000 Hz.

Firstly, a sound signal test experiment with a fixed frequency was carried out. The SPL of the test sound was 100 dB, and the frequencies were from 3000 to 9000 Hz. Signal acquisition was finished by a self-developed signal acquisition circuit. Data analysis was completed by MATLAB 2016(b). The sampling number was 10^5^, which corresponded to a 5 ms sound signal. Figure 13 shows the output voltage of a 3000 Hz sound signal with sampling points of 10^5^. The test result showed that the system has a good response to the signal at 3000 Hz. For the sound signal of 3000 Hz, the peak-to-peak value of the system was 345.19 mV, and the sensitivity was 172.60 mV/Pa.

**Figure 14:**
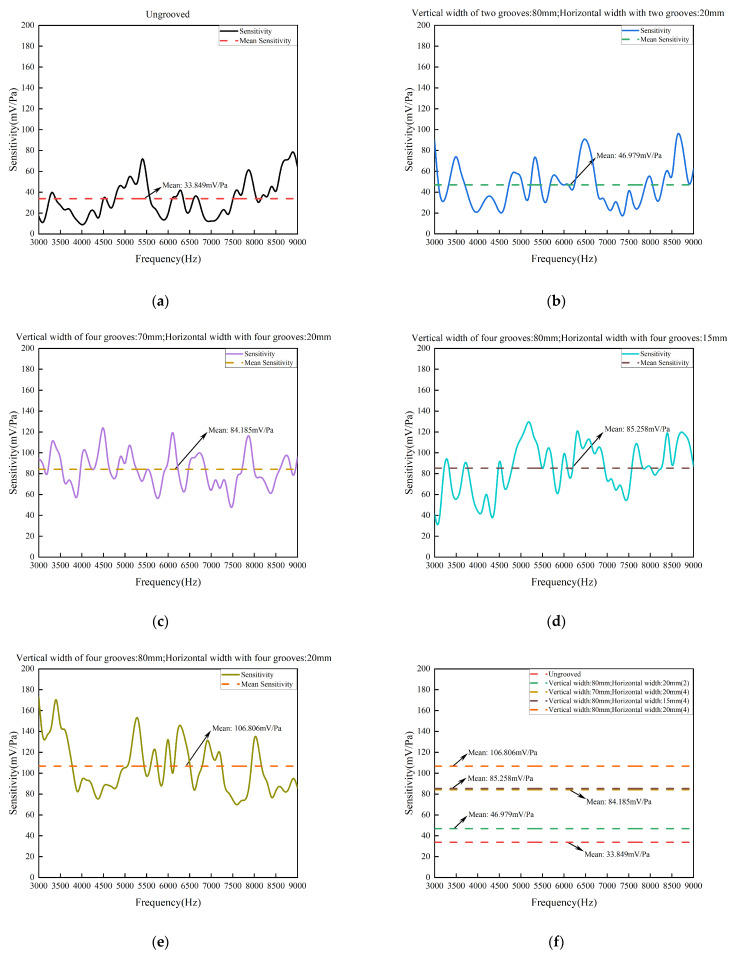
The sensitivity with different pickups: (**a**) the sensitivity of the ungrooved pickup, (**b**) the sensitivity of the two-groove pickup with a vertical width of 80 mm and a horizontal width of 20 mm, (**c**) the sensitivity of the four-groove pickup with a vertical width of 70 mm and a horizontal width of 20 mm, (**d**) the sensitivity of the four-groove pickup with a vertical width of 80 mm and a horizontal width of 15 mm, (**e**) the sensitivity of the four-groove pickup with a vertical width of 80 mm and a horizontal width of 20 mm, and (**f**) the mean sensitivity with different pickups.

As shown in Figure 14 and Table 2, the variation of the resolution was analyzed to verify the response of the system at different frequencies. The frequency interval was 100 Hz. The average peak-to-peak sensitivity of the system was 33.849 mV/Pa with the ungrooved pickup. The average peak-to-peak sensitivity of the system was 46.979 mV/Pa with the two-groove pickup with a vertical width of 80 mm and a horizontal width of 20 mm. The average peak-to-peak sensitivity of the system was 84.185 mV/Pa with the four-groove pickup with a vertical width of 70 mm and a horizontal width of 20 mm. The average peak-to-peak sensitivity of the system was 85.258 mV/Pa with the four-groove pickup with a vertical width of 80 mm and a horizontal width of 15 mm. The average peak-to-peak sensitivity of the system was 106.806 mV/Pa with the four-groove pickup with a vertical width of 80 mm and a horizontal width of 20 mm. The average peak-to-peak sensitivity of the grooved pickups increased by 38.79%, 148.71%, 151.88%, 215.54%, respectively. The experiment showed that sensitivity improves significantly by the four-groove pickup with a vertical width of 80 mm and a horizontal width of 20 mm, which is consistent with the simulation results.

**Figure 15:**
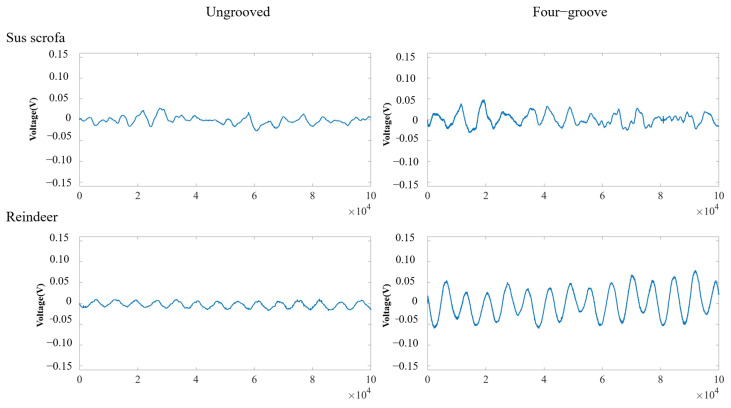

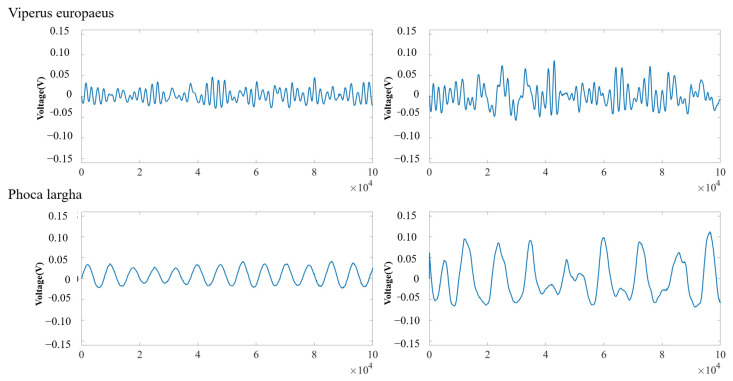
The test results of ungrooved and grooved pickups with different animals.

Furthermore, measurements of natural sounds in a laboratory environment were completed. As shown in Figure 15, the sounds of sus scrofa, reindeer, viperus europaeus, and phoca largha were tested by the improved system. As shown in Table 3, the grooved pickup showed different degrees of improvement compared to the ungrooved pickup. The peak-to-peak value of sus scrofa was 0.055 V with the ungrooved pickup and increased to 0.08 V with the four-groove pickup. The peak-to-peak value of reindeer was 0.029 V with the ungrooved pickup and increased to 0.140 V with the four-groove pickup. The peak-to-peak value of viperus europaeus was 0.076 V with the ungrooved pickup and increased to 0.145 V with the four-groove pickup. The peak-to-peak value of phoca largha was 0.065 V with the ungrooved pickup and increased to 0.182 V with the four-groove pickup. The peak-to-peak value of sus scrofa, reindeer, viperus europaeus, and phoca largha increased by 45.45%, 382.76%, 90.79%, and 180.00%, respectively.

## 5. Conclusions

In summary, a new pickup was proposed and optimized to improve the sound response sensitivity of the system. The key conclusions are as follows.

(1) Theoretical calculation and simulation analysis were carried out, and the parameters of the pickup were determined.

(2) Aluminum was chosen as the material of the pickup. The size of the cylinder was set to a height of 120 mm, a diameter of 110 mm, and a thickness of 3 mm. The size of grooving was set to a vertical width of 80 mm and a horizontal width of 20 mm.

(3) Experimental results showed that the average peak-to-peak sensitivity of the pickup optimized for grooving increases by 215.54% to 106.806 mV/Pa compared to the ungrooved pickup in the frequency range of 3000–9000 Hz. The calls of different wild animals can be distinguished, and the response sensitivity of wild animals with different frequencies improves in different degrees.

These results show that the system has a prospect of application in areas such as wildlife nature reserves to monitor the situation of invasion. In further research, continuous optimization of the slotted structure and continuous improvement of the consistency of the system response to different frequencies will be the key to optimize the performance of the system.

## Figures and Tables

**Table 1:** The relationship between material and maximum strain.

Material	Maximum Strain
Iron	9.673 × 10^−11^
Stainless steel	6.024 × 10^−11^
Copper	1.100 × 10^−10^
Aluminum	1.706 × 10^−10^
Titanium	1.153 × 10^−10^

**Table 2:** The average peak-to-peak sensitivity of the pickups.

Sizes of the Pickups	Average Peak-To-Peak Sensitivity of the Ungrooved Pickup (mV/Pa)	Average Peak-to-Peak Sensitivity of Grooved Pickups (mV/Pa)	Percentage of Growth
Ungrooved	33.849	/	/
V: 80 mm; H: 20 mm (2)	33.849	46.979	38.79%
V: 70 mm; H: 20 mm (4)	33.849	84.185	148.71%
V: 80 mm; H: 15 mm (4)	33.849	85.258	151.88%
V: 80 mm; H: 20 mm (4)	33.849	106.806	215.54%

**Table 3:** The peak-to-peak value of the ungrooved pickup and the four-groove pickup.

Natural Sounds	Peak-To-Peak Value of the Ungrooved Pickup (V)	Peak-To-Peak Value of the Four-Groove Pickup (V)	Percentage of Growth
Sus scrofa	0.055	0.080	45.45%
Reindeer	0.029	0.140	382.76%
Viperus europaeus	0.076	0.145	90.79%
Phoca largha	0.065	0.182	180.00%

## Data Availability

Data are contained within the article.
